# Flexible ultra-sensitive and resistive NO_2_ gas sensor based on nanostructured Zn_(*x*)_Fe_(1−*x*)2_O_4_

**DOI:** 10.1039/c7ra10478b

**Published:** 2018-01-17

**Authors:** Solleti Goutham, Kishor Kumar Sadasivuni, Devarai Santhosh Kumar, Kalagadda Venkateswara Rao

**Affiliations:** Nano Electronics Laboratory, Centre for Nano Science and Technology, JNT University Hyderabad Kukatpally Hyderabad-500085 Telangana State India kalagadda2003@jntuh.ac.in +91 9440858664; Center for Advanced Materials, Qatar University P. O. Box 2713 Doha Qatar; Department of Chemical Engineering, IIT – Hyderabad Kandi-502285 Telangana India

## Abstract

Low concentration gas detection, rapid response time and low working temperature are anticipated for a varied range of toxic gas detection applications. Conversely, the existing gas sensors suffer mostly from a high working temperature along with a slow response at low concentrations of analytes. Here, we report an ultrasensitive flexible nanostructured Zn_(*x*)_Fe_(1−*x*)2_O_4_ (*x* = 0.1, 0.5 and 0.9) based chemiresistive sensor for nitrogen dioxide (NO_2_) detection. We evince that the prepared flexible sensor Zn_(0.5)_Fe_(0.5)2_O_4_ has detection potential as low as 5 ppm at a working temperature of 90 °C in a short phase. Further, the Zn_(0.5)_Fe_(0.5)2_O_4_ sensor exhibits excellent selectivity, stability and repeatability. The optimized sensor sensing characteristics can be helpful in tremendous development of foldable mobile devices for environmental monitoring, protection and control.

## Abstract 2

Low concentration gas detection, rapid response time and low working temperature are anticipated for a varied range of toxic gas detection applications.

## Graphical abstract

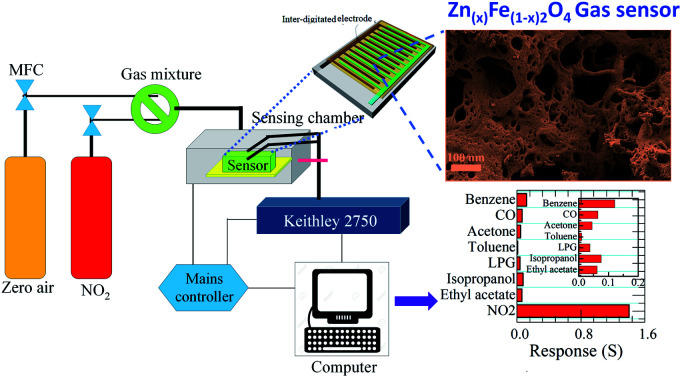

## 1. Introduction

There is a high interest in the discovery of novel nanomaterials in order to develop rapid response and extremely sensitive solid-state gas sensors. Semiconductor metal oxide materials are an alternative to conventional sensing materials due to their exceptional characteristics of easy synthesis, cost-effectiveness and low power consumption. These materials are the best candidates to detect poisonous, toxic, flammable, explosive and harmful gases. The gas sensor working principle involves the surface adsorbed atmospheric oxygen species interaction with analyte gas molecules, leading to redox reactions on the semiconductor, such as ZnO,^1,2^ WO_3_,^3,4^ In_2_O_3_,^5,6^ SnO_2_ (ref. 7–10) and Fe_2_O_3_ (ref. 11,12) (n-type), and NiO^13^ and CoO_3_O_4_ (ref. 14) (p-type) gas sensors. In sensing application crucial roles are performed by properties regarding oxides of semiconductor like pores, grain size, crystalline size, film thickness, layers, and surface to volume ratio.^15,16^ Furthermore, if the prepared sensing material is porous, the targeted analyte molecules can easily penetrate the material and react with the total volume of the material by enhancing the sensor response tremendously.^17^ Thus, sufficient attention has been concentrated on controlling the structural and morphological parameters of nanomaterials with high-energy surfaces^18,19^ and decreased crystalline size,^20^ which are gaining special attention, such as oxide composites, core–shell heterostructure nanotubes,^21,22^ 3D structures^23,24^ and doping.^25^

Spinel ferrites are the basic functional material used in a variety of cutting-edge technological applications because it is exceptionally good catalyst and has simple synthesis. Additionally it is very economical and eco-friendly in nature.^26^ ZnFe_2_O_4_ has been widely used with lithium-ion batteries as the anode materials for the past few years. Nanostructured ZnFe_2_O_4_ is a gas sensing material with rapid response and excellent selectivity towards oxidizing and reducing gases. The scientists working on the intrinsic association between shape, structure and gas sensing characteristics have produced essential adaptable synthetic strategies, where these properties of ZnFe_2_O_4_ can be tailored with designed functionalities. In this regard, the preparation of nanostructured ZnFe_2_O_4_ with exclusive microstructures is escalating its possible gas sensor applications.

In the present paper, a simple sol–gel auto combustion method was used to synthesize nanostructured Zn_(*x*)_Fe_(1−*x*)2_O_4_ (*x* = 0.1, 0.5 and 0.9).^27^ As a result, a large specific surface area pore size was exhibited by the prepared ZnFe_2_O_4_ materials. A flexible device was fabricated by using a simple drop drying technique. Additionally, NO_2_ gas sensing characteristics were investigated with various working temperatures. We proved that a nanostructured flexible Zn_(*x*)_Fe_(1−*x*)2_O_4_ (*x* = 0.5) based sensor shows a high response at an operating temperature of 90 °C, with excellent selectivity, good stability and reproducibility.

## 2. Experimental section

### 2.1 Synthesis of nanostructured Zn_(*x*)_Fe_(1−*x*)2_O_4_ (*x* = 0.1, 0.5 and 0.9)

A sol–gel-auto combustion technique was employed to prepare nanostructured Zn_(*x*)_Fe_(1−*x*)2_O_4_ (*x* = 0.1, 0.5 and 0.9).^27^ In this method, the exothermic reaction of xerogel, which is an aqueous solution of metal nitrates (zinc nitrate and iron nitrate) and fuel (glycine) was carried out. All the reagents were of analytical grade from Sigma-Aldrich, USA. An appropriate amount of nitrates and fuel were dissolved in distilled water under constant stirring at 80 °C according to the stoichiometric composition of the fuel to oxidizer ratio. After 25–30 min a brown colored thick gel was formed. The obtained solution was placed on a hot plate at 180 °C to initiate the combustion, then it was ignited to form a lightweight powder and annealed at 650 °C for 5 h.

### 2.2 Characterization

The structural analysis of the nanostructured Zn_(*x*)_Fe_(1−*x*)2_O_4_ (*x* = 0.1, 0.5 and 0.9) powder was made with a Bruker-D8 X-ray diffractometer (XRD) using Cu Kα_1_ radiation. The optical property of absorbance was calculated by a UV-visible double beam spectrophotometer (Systronic-2203). Fourier transform infrared spectroscopy (FT-IR) (PerkinElmer L160000A) in the wavelength range of 500–4000 cm^−1^ was also used for the structural elucidation. The morphology and elemental composition were observed by a Carl Zeiss (Merlin compact 60-27) field emission scanning electron microscope (EDX and FESEM). Particle size and morphology were further confirmed by transmission electron microscopy (TEM) (Philips, Holland TEM instrument) operated at an accelerating voltage of 120 kV. Resistance and voltage were measured using the Keithley multimeter (2750).

### 2.3 Device fabrication and construction of in-house sensor testing unit

**Fig. 1:**
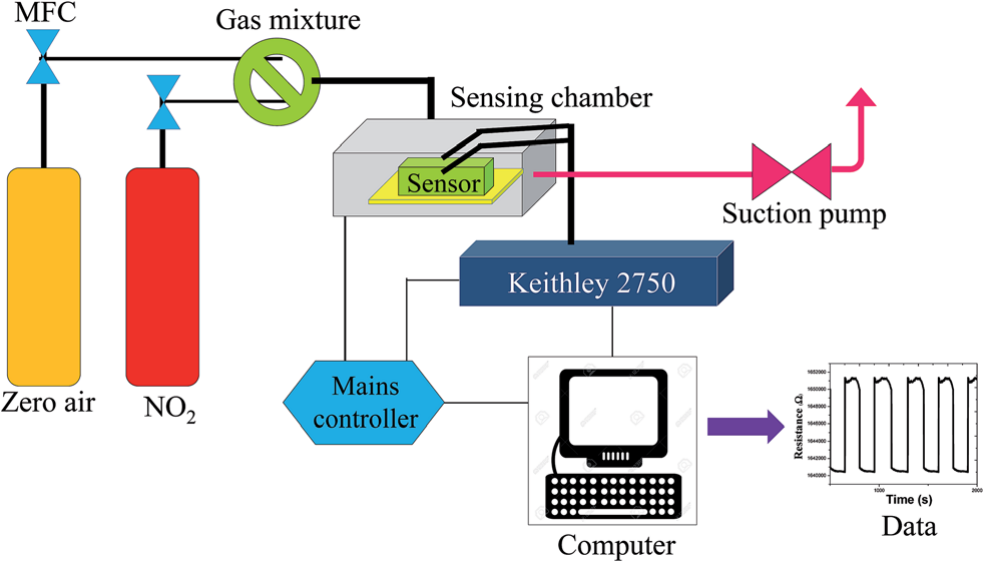
Schematic of in-house setup for gas sensing.

The samples were coated with a drop drying method on flexible electrodes prior to testing, which can be described as follows. Initially, an approximate amount of the as-prepared Zn_(*x*)_Fe_(1−*x*)2_O_4_ (*x* = 0.1, 0.5 and 0.9) nanostructured powder was mixed with dimethylformamide to prepare the homogeneous paste and coated onto flexible pre-patterned interdigitated electrodes (IDEs). This was then allowed to dry at room temperature, and the device was calcinated at 150 °C for 3 h to enhance its stability. Finally, the fabricated device was connected to a Keithley multimeter (2750) in an in-house dynamic gas sensing setup. The sensing examination of the developed gas sensor was examined by a sensor testing unit, which was explained comprehensively in our earlier paper.^28^ The construction of the in-house sensing setup is schematically demonstrated in Fig. 1. The sensor response was calculated as *S* = (*R*_a_ − *R*_g_)/*R*_a_ for oxidizing gases or (*R*_g_ − *R*_a_)/*R*_g_ for reducing gases, where *R*_a_ is the resistance value in absence of air and *R*_g_ is the resistance in the presence of the analyte gas. The sensor was analyzed in both flat and bending position to demonstrate the flexibility of the sensor. A bending angle of 60° was used to determine the flexibility.

## 3. Results and discussion

### 3.1 Structural and morphological characteristics

**Fig. 2:**
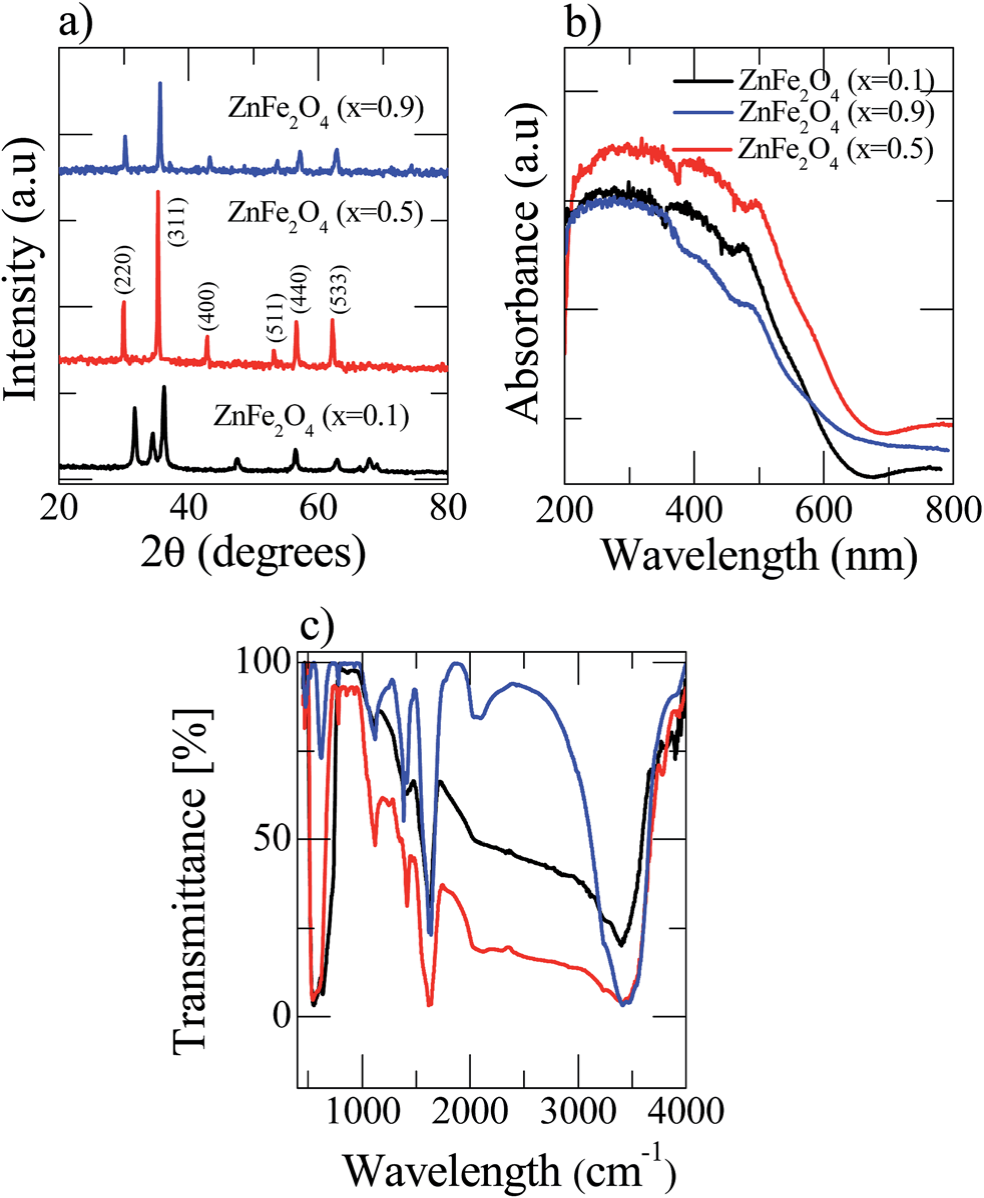
Room temperature analysis of the three as-prepared Zn_(*x*)_Fe_(1−*x*)2_O_4_ (*x* = 0.1, 0.5 and 0.9) samples (a) XRD patterns (b) UV-visible spectroscopy and (c) Fourier transform infrared spectroscopy.

The X-ray diffraction (XRD) spectra of the pre-synthesized nanostructured Zn_(*x*)_Fe_(1−*x*)2_O_4_ (*x* = 0.1, 0.5 and 0.9) is shown in Fig. 2a. The Zn_(*x*)_Fe_(1−*x*)2_O_4_ (*x* = 0.5) deflection peaks were in good agreement with the standard JCPDS no. 89-1012, which shows the product is highly pure and has no other impurities. This indicates that metal nitrates were fully transformed into ZnFe_2_O_4_ at 650 °C. Whereas in the case of the Zn_(*x*)_Fe_(1−*x*)2_O_4_ (*x* = 0.1 and 0.9) materials, due to their compositional variations, a slight peak shift was noticed (Fig. 2a). Nanostructured Zn_(*x*)_Fe_(1−*x*)2_O_4_ (*x* = 0.5) deflection peaks were comparatively broadened, indicating its small crystallite size. The average crystal sizes of nanostructured Zn_(*x*)_Fe_(1−*x*)2_O_4_ (*x* = 0.1, 0.5 and 0.9) are about 21.5 nm, 16.8 nm and 18.7 nm, respectively. The crystalline size was estimated by the Debye–Scherrer formula *D* = 0.89*λ*/*β* cos *θ* (where *λ* = 1.54060 Å, *θ* is the Bragg angle and *β* is the peak full width at half maximum).

The UV-vis spectra of the Zn_(*x*)_Fe_(1−*x*)2_O_4_ (*x* = 0.1, 0.5 and 0.9) are shown in Fig. 2b and a strong absorption was observed for all the prepared samples. Zn_(*x*)_Fe_(1−*x*)2_O_4_ (*x* = 0.5) exhibits peak shift from UV to visible light region and reveals the nanostructured Zn_(*x*)_Fe_(1−*x*)2_O_4_ (*x* = 0.5) having a higher efficiency to absorb visible light than Zn_(*x*)_Fe_(1−*x*)2_O_4_ (*x* = 0.1 and 0.9). Moreover, the absorption spectrum of the Zn_(*x*)_Fe_(1−*x*)2_O_4_ (*x* = 0.5) material contained both regions (UV and visible) of Zn_(*x*)_Fe_(1−*x*)2_O_4_ (*x* = 0.1 and 0.9). These results show that an equal composition of precursor material causes expansion and enhancement of the photoresponse to the visible region. Hence, we observe that the photocatalytic activity of the prepared Zn_(*x*)_Fe_(1−*x*)2_O_4_ (*x* = 0.5) was superior to that of Zn_(*x*)_Fe_(1−*x*)2_O_4_ (*x* = 0.1 and 0.9) in visible light.

A Fourier transform infrared (FTIR) spectrophotometer was used to analyze the Zn_(*x*)_Fe_(1−*x*)2_O_4_ (*x* = 0.1, 0.5 and 0.9) nanostructured materials in the range from 500 to 4000 cm^−1^ and the results are shown in Fig. 2c. The FTIR spectra of all three samples show the peak at 1274 cm^−1^ represented =C–H in-plane stretching. The peak at 923 cm^−1^ is from C–C out-of-plane stretching vibrations^29^ and the broad band observed at 3430 cm^−1^ is related to O–H vibrations. Peaks at vibrations around 500 cm^−1^ are attributed to Fe–O and Zn–O vibrations.^30^

**Fig. 3:**
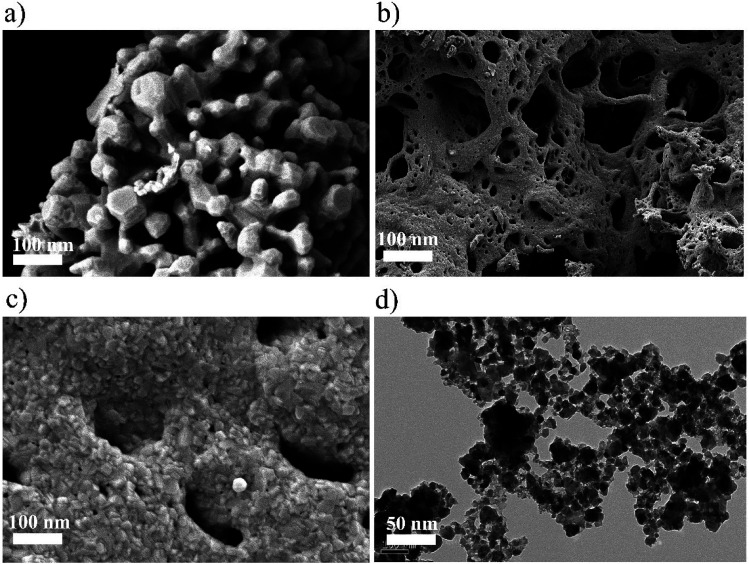
(a) FESEM image of Zn_(0.1)_Fe_(0.9)2_O_4_ (b) FESEM image of Zn_(0.5)_Fe_(0.5)2_O_4_ (c) FESEM image of Zn_(0.9)_Fe_(0.1)2_O_4_ and (d) typical HRTEM image of Zn_(0.5)_Fe_(0.5)2_O_4_.

**Fig. 4:**
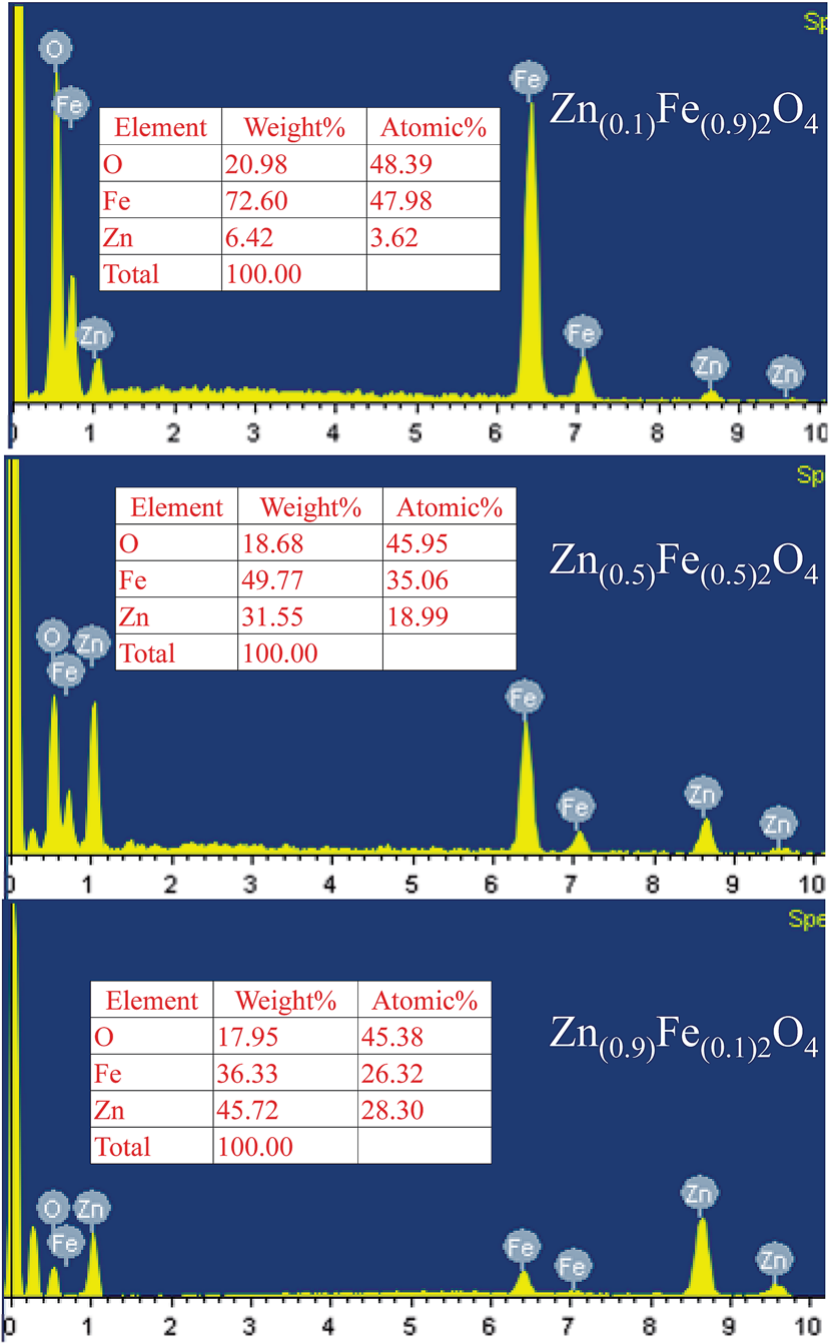
EDS spectrum and composition data of the Zn_(*x*)_Fe_(1−*x*)2_O_4_ (*x* = 0.1, 0.5 and 0.9) samples.

The nanostructured Zn_(*x*)_Fe_(1−*x*)2_O_4_ (*x* = 0.1, 0.5 and 0.9) morphologies are shown in Fig. 3a–c and the structures were analyzed by field emission scanning electron microscopy (FESEM). All the samples exhibited nanocrystalline and mixed shaped cellular structures. A qualitative examination reveals that Zn_(*x*)_Fe_(1−*x*)2_O_4_ (*x* = 0.5) material is smaller, with minute interstices of a more distinctive and uniform nature than those of the Zn_(*x*)_Fe_(1−*x*)2_O_4_ (*x* = 0.1 and 0.9) materials. An equal amount of precursor, *i.e.*, Zn_(0.5)_Fe_(0.5)2_O_4_ (Fig. 3b) displayed a necked type particle cluster and porous nature. The crystalline sizes of the samples analyzed by FE-SEM exhibit an average of 29.3 nm, 20.4 nm and 22.5 nm for nanostructured Zn_(*x*)_Fe_(1−*x*)2_O_4_ (*x* = 0.1, 0.5 and 0.9), respectively. These values were compared with XRD measurements and where observed to be in good agreement. The typical HRTEM of the nanostructured Zn_(*x*)_Fe_(1−*x*)2_O_4_ (*x* = 0.5) is illustrated in Fig. 3d. The size and structure of the material were in harmony with the FESEM results. These results confirm a massive amount of particles are of a nano size and assembled to form a spherical structure. Fig. 4 represents the elemental analysis of the nanostructured Zn_(*x*)_Fe_(1−*x*)2_O_4_ (*x* = 0.1, 0.5 and 0.9) materials and evidences the presence of Zn, Fe and O.

### 3.2 Gas sensing properties

**Fig. 5:**
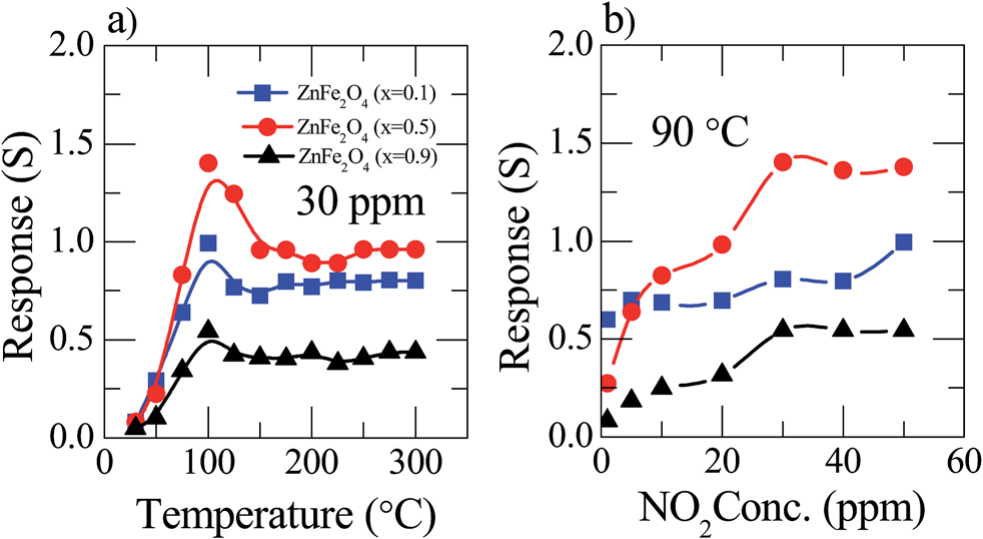
Sensor response of Zn_(*x*)_Fe_(1−*x*)2_O_4_ (*x* = 0.1, 0.5 and 0.9) of (a) to 30 ppm NO_2_ gas at different operating temperatures (b) constant 90 °C temperature at various NO_2_ gas concentration.

The present study deals with the advantages of the pre-synthesized nanostructured materials Zn_(*x*)_Fe_(1−*x*)2_O_4_ (*x* = 0.1, 0.5 and 0.9) as NO_2_ sensing substances and their characteristics. In a chemiresistive gas sensor, the sensitivity mainly depends upon the operating temperature. Thus, the responses of Zn_(*x*)_Fe_(1−*x*)2_O_4_ (*x* = 0.1, 0.5 and 0.9) based sensors were measured by changing the temperature from room temperature to 300 °C at a constant gas concentration of 30 ppm and the related examined results are illustrated in Fig. 5a. In the figure, it is clearly shown that cone shape curves represent that the initial NO_2_ response was enhanced with operating temperature and reaches its highest value for about 90 °C, and afterwards reduces slowly. The threshold temperature for the sensor was observed at 90 °C. Among all three prepared samples, Zn_(*x*)_Fe_(1−*x*)2_O_4_ (*x* = 0.1, 0.5 and 0.9), the maximum response was observed for the equal amount of precursor material *i.e.*, Zn_(*x*)_Fe_(1−*x*)2_O_4_ (*x* = 0.5). The obtained increase–decrease result responses can be described as follows: at room temperature, NO_2_ gas molecules partially interact with the surface absorbed atmospheric oxygen molecules, which gives a lower response. Whereas, with increasing the operating temperature the rate of reaction becomes higher and there is an increase in the oxygen ions on the sensor surface from the absorbed atmospheric oxygen species (O_2(gas)_ → O_2(ads)_ → O_2(ads)_^−^ → 2O_(ads)_^−^) responsible for the maximum response. From the result, 90 °C is the optimum working temperature for the selected nanostructured Zn_(*x*)_Fe_(1−*x*)2_O_4_ (*x* = 0.5). In the case of Zn_(*x*)_Fe_(1−*x*)2_O_4_ (*x* = 0.1 and 0.9), the sensor response observed was 0.74 and 0.54%, respectively, for 90 °C at 30 ppm and comparatively these sensitivity results were a much smaller response than the sensor response (1.41%) of the equal ratio precursor (Zn_(0.5)_Fe_(0.5)2_O_4_). The reason behind this kind of behavior might be due to the Zn_(0.5)_Fe_(0.5)2_O_4_ sensor having enough porosity so that NO_2_ gas can easily penetrate inside the sensing material throughout the surface. The nanostructured Zn_(*x*)_Fe_(1−*x*)2_O_4_ (*x* = 0.1 and 0.9) sensor material has a low porosity compared to the Zn_(0.5)_Fe_(0.5)2_O_4_ materials.

The response of the Zn_(*x*)_Fe_(1−*x*)2_O_4_ (*x* = 0.1, 0.5 and 0.9) sensor variations along with the NO_2_ gas dilutions was examined at an optimum working temperature (Fig. 5b). It is observed that as the gas concentration value increases slowly from 1 ppm, the sensor response increased up to 30 ppm and then saturated. This kind of sensing behavior can be estimated as follows: a very low surface interaction at a minute gas concentration leads to a low sensor response. The sensitivity increased stepwise when the gas concentration increased to create an enhanced sensor surface interaction, leading to a rising response at a critical concentration, *i.e.* 30 ppm. Followed by a increased NO_2_ gas concentration, the sensor surface was fully covered by and therefore had no possibility to react with new molecules due to the saturation level.

**Fig. 6:**
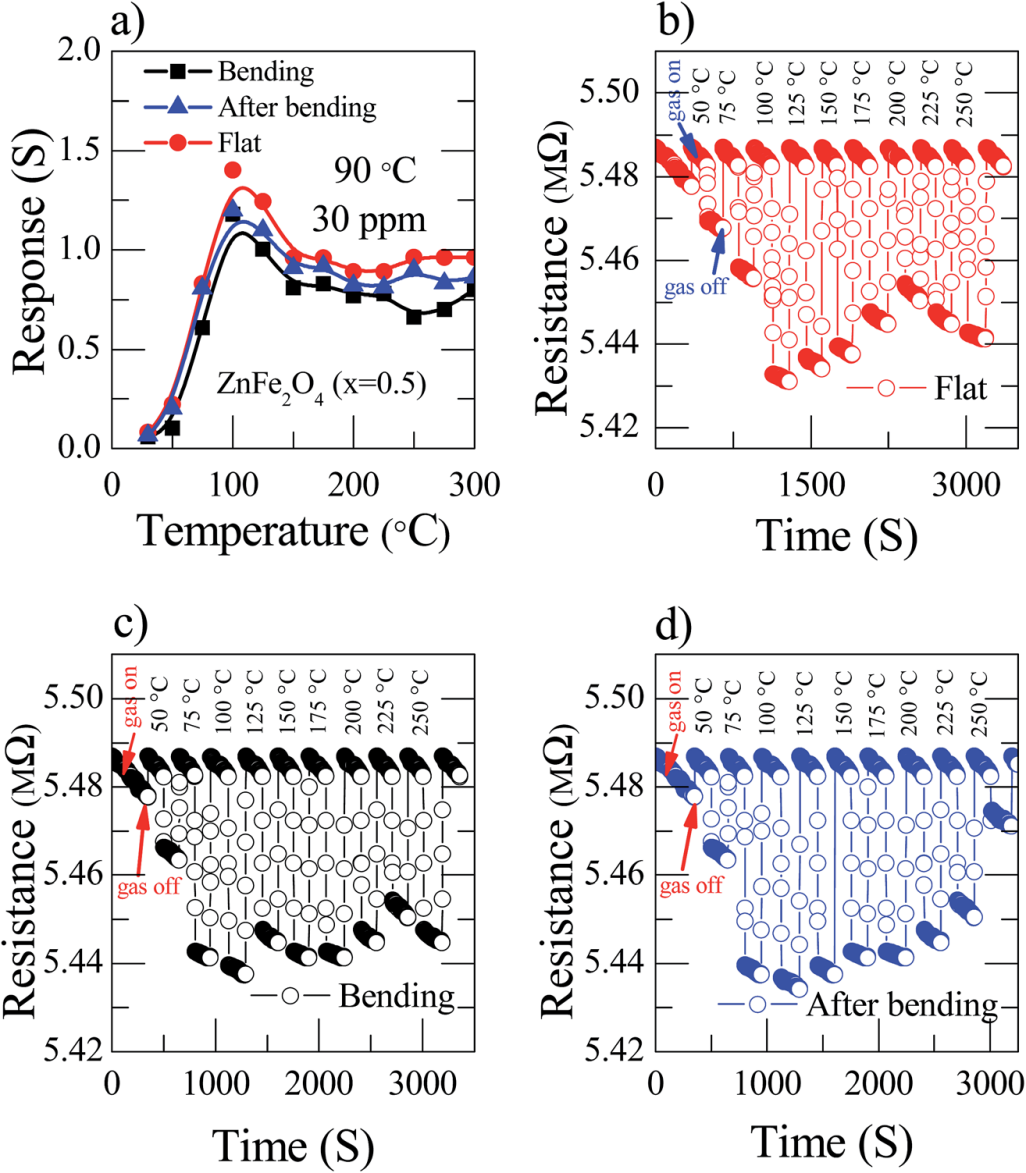
(a) Zn_(0.5)_Fe_(0.5)2_O_4_ sensor response at 30 ppm NO_2_ gas at 90 °C temperature and different bending conditions (red color stands for flat, black for bending and blue for after bending) (b–d) dynamic response of the recovery behavior of the Zn_(0.5)_Fe_(0.5)2_O_4_ sensors towards 30 ppm NO_2_ gas at 90 °C flat, bending and after bending conditions, respectively.

The tremendous fabricated current device is solely transparent and mechanically flexible. To investigate the nanostructured Zn_(0.5)_Fe_(0.5)2_O_4_ sensor, flexibility properties were measured in flat (red), bending (black) and after bending (blue) conditions with a bending radius of 60° for 30 ppm NO_2_ at 90 °C. Fig. 6a illustrates the sensor response based on flexibility and temperature variations. The response of the sensor device decreased moderately while bending due to the reduction of the sensitive surface area. Fascinatingly, the fabricated device did not deteriorate considerably after these mechanical transformations. Moreover, the few dynamic cycles of the response–recovery of the flexible sensor shown in Fig. 6b–d depicted different conditions at various temperatures for 30 ppm NO_2_.

**Fig. 7:**
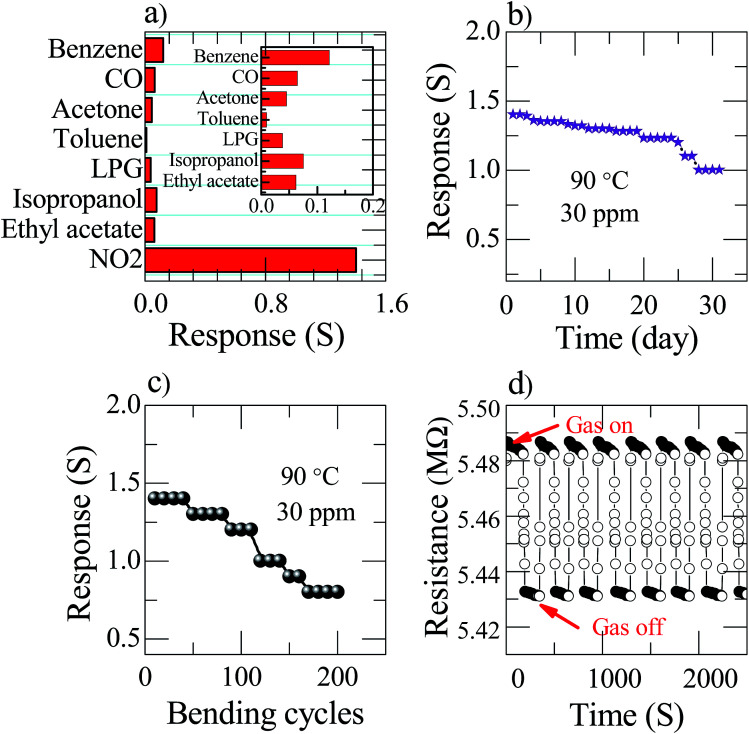
Zn_(0.5)_Fe_(0.5)2_O_4_ sensor at an operating temperature of 90 °C for 30 ppm (a) selectivity. The inset represents the response to various analytes (small scale) (b) stability (c) bending test (d) reproducibility.

The nanostructured Zn_(0.5)_Fe_(0.5)2_O_4_ sensor selectivity was studied by exposing different analytes, such as benzene, carbon monoxide, acetone, toluene, LPG, isopropanol and ethyl acetate, as shown in Fig. 7a. The concentration of all the analytes was maintained constant, *i.e.* 30 ppm at 90 °C. The sensor response against NO_2_ was remarkably higher than against other analytes. This analysis provides the information regarding the high selectivity of the sensor towards NO_2_. This is due to the chemisorbed atmospheric oxygen present on the sensor surface effectively initiating a high response towards NO_2_. In the practical application of gas sensors, long-term stability is one of the essential characteristic parameters. Therefore, the response of the prepared flexible Zn_(0.5)_Fe_(0.5)2_O_4_ sensor to 30 ppm of NO_2_ at a temperature of 90 °C was examined for 30 days as illustrated in Fig. 7b. In that period of time, the sensor response was recorded with only a little fluctuation. Therefore, fabrication of attractive and promising sensor with an excellent durability is possible with this technique. These sensors are very useful in a direct industrial application. Bending tests were implemented on the sensor during the test to understand the flexibility of the sensor. After two hundred repeats of bending of the prepared flexible sensor, the device did not have a large deviation of response (Fig. 7c). To examine the repeatability of the flexible sensor, the Zn_(0.5)_Fe_(0.5)2_O_4_ device were exposed to seven cycles of 30 ppm NO_2_ and the dynamic resistance responses are shown in Fig. 7d. The test revealed that the sensor response is constantly maintained after several exposure cycles. From the obtained results, the sensor shows good reproducibility for a long time, which confirms the stability of the prepared gas sensor towards NO_2_.

### 3.3 Gas sensing mechanism

**Fig. 8:**
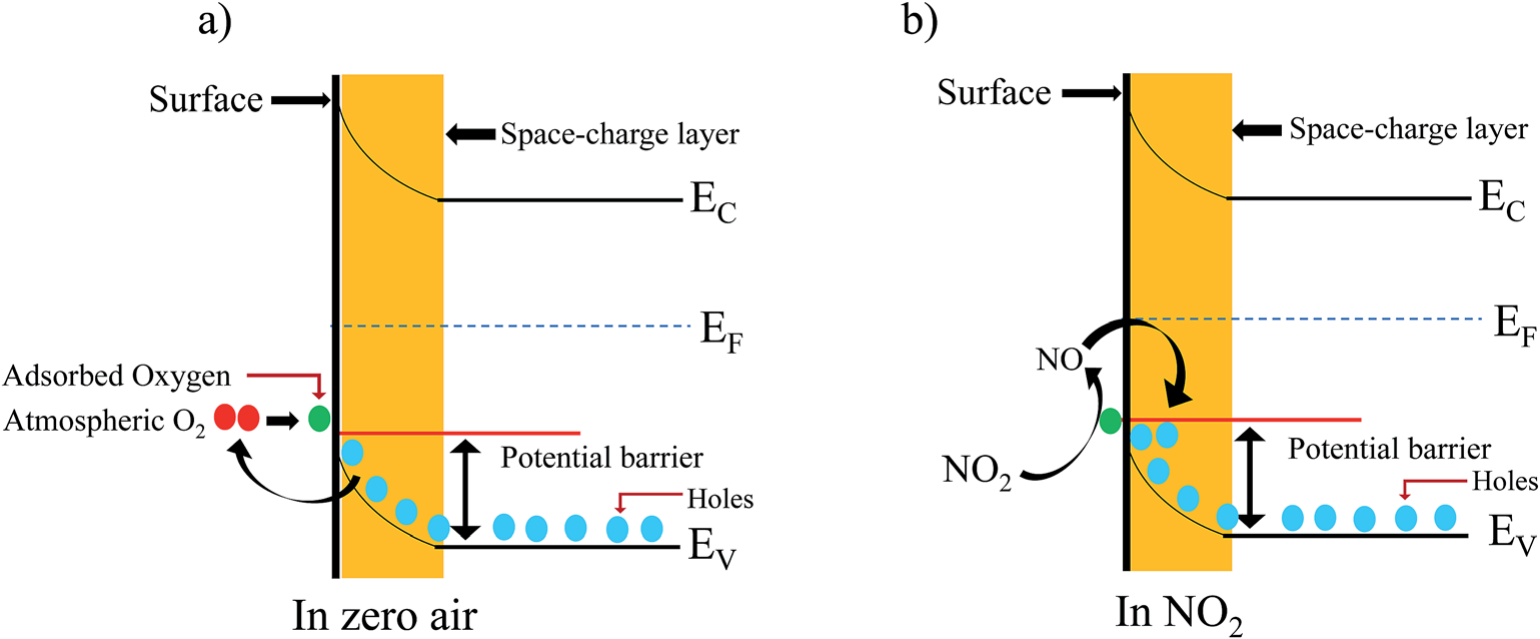
(a and b) Schematic illustration of the air and NO_2_ mechanisms for nanostructured ZnFe_2_O_4_.

A well-known p-type semiconductor oxide^31^ such as ZnFe_2_O_4_, works as a sensor and the functioning mechanism involved is based on the change in resistance of the atmospheric oxygen molecule chemisorption on the surface of the sensing material.^32,33^ ZnFe_2_O_4_ contains holes as the major charge carrier. Initially, when the flexible ZnFe_2_O_4_ sensor is exposed to zero air, the atmospheric oxygen molecules in the form of O_2(ads)_^−^, O_(ads)_^−^ and O^2−^_(ads)_ adsorb on the surface of the sensor. The sensor resistance increases due to the formation of a thick electron space charge layer on the surface (Fig. 8a). Followed by the exposure to an oxidizing gas, *i.e.* NO_2_ (electron accepting), molecules interact with adsorbed oxygen molecules, which leads to a decrease in resistance (Fig. 8b). The flexible ZnFe_2_O_4_ gas sensor performance towards NO_2_ gas is compared with previously reported ZnFe_2_O_4_ gas sensor for various analytes as listed in Table 1.

**Table 1:** The present study compared with the literature on ZnFe_2_O_4_ based NO_2_ gas sensor

S. No.	Material	Substrate	Operating temperature (°C)	Gas concentration	Reference
1	WO_3_	Hard	27	320 ppb	34
2	Graphene	Hard	27	25 ppm	35
3	ZnO	Hard	27	16 ppm	36
4	S/graphene	Hard	27	100 ppm	37
5	CoTa_2_O_6_	Hard	650	100 ppm	38
6	Ce/NiO	Hard	150	40 ppm	39
7	(rGO)–In_2_O_3_	Flexible	150	500 ppb	40
8	Zn_(0.5)_Fe_(0.5)2_O_4_	Flexible	90	5 ppm	Present work

## 4. Conclusion

In summary, a sol–gel auto combustion method was used for the preparation of nanostructured flexible Zn_(*x*)_Fe_(1−*x*)2_O_4_ (*x* = 0.1, 0.5 and 0.9), which were coated on a pre-patterned flexible electrode by a simple drop drying process and heated afterwards. The synthesized material was examined as a sensing material for the possible chemiresistive gas sensing application. It was found that the equal concentration of precursor material (Zn_(*x*)_Fe_(1−*x*)2_O_4_ (*x* = 0.5)) used in device exhibited an ultra-high sensing performance and excellent long-term stability, selectivity and reproducibility towards 5 ppm of NO_2_ at a working temperature of 90 °C.

## Conflicts of interest

There are no conflicts to declare.

## Supplementary Material
